# Supplementary figures and images for: Phosphorylation bar-coding of free fatty acid receptor 2 is generated in a tissue-specific manner
Source: eLife. 2023 Dec 12;12:RP91861. doi: 10.7554/eLife.91861 (PMC10715726; doi:10.7554/eLife.91861)

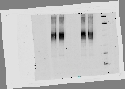

Supplement: Figure 2—source data 1. [file elife-91861-fig2-data1.zip › 0003595_04/0003595_04_TH.jpg]

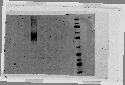

Supplement: Figure 2—source data 2. [file elife-91861-fig2-data2.zip › 0003593_03/0003593_03_TH.jpg]

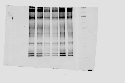

Supplement: Figure 2—source data 3. [file elife-91861-fig2-data3.zip › 0003594_01/0003594_01_TH.jpg]

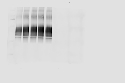

Supplement: Figure 2—source data 5. [file elife-91861-fig2-data5.zip › 0004559_01/0004559_01_TH.jpg]

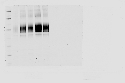

Supplement: Figure 2—source data 6. [file elife-91861-fig2-data6.zip › 0004557_01/0004557_01_TH.jpg]

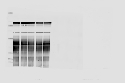

Supplement: Figure 2—source data 7. [file elife-91861-fig2-data7.zip › 0004555_01/0004555_01_TH.jpg]

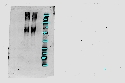

Supplement: Figure 3—source data 1. [file elife-91861-fig3-data1.zip › 0003310_01/0003310_01_TH.jpg]

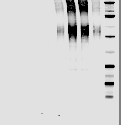

Supplement: Figure 3—source data 2. [file elife-91861-fig3-data2.zip › 0003308_09/0003308_09_TH.jpg]

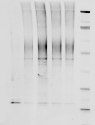

Supplement: Figure 3—source data 3. [file elife-91861-fig3-data3.zip › 0003311_06/0003311_06_TH.jpg]

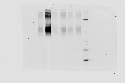

Supplement: Figure 3—source data 4. [file elife-91861-fig3-data4.zip › 0002962_03/0002962_03_TH.jpg]

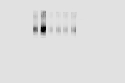

Supplement: Figure 3—source data 5. [file elife-91861-fig3-data5.zip › 0002961_02/0002961_02_TH.jpg]

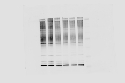

Supplement: Figure 3—source data 6. [file elife-91861-fig3-data6.zip › 0002963_03/0002963_03_TH.jpg]

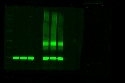

Supplement: Figure 4—source data 1. [file elife-91861-fig4-data1.zip › 0003729_01/0003729_01_TH.jpg]

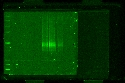

Supplement: Figure 4—source data 2. [file elife-91861-fig4-data2.zip › 0003629_01/0003629_01_TH.jpg]

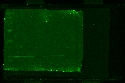

Supplement: Figure 4—source data 3. [file elife-91861-fig4-data3.zip › 0003741_01/0003741_01_TH.jpg]

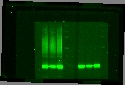

Supplement: Figure 5—source data 1. [file elife-91861-fig5-data1.zip › 0003276_02/0003276_02_TH.jpg]

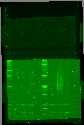

Supplement: Figure 5—source data 2. [file elife-91861-fig5-data2.zip › 0003278_02/0003278_02_TH.jpg]

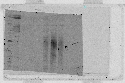

Supplement: Figure 5—source data 3. [file elife-91861-fig5-data3.zip › 0003873_01/0003873_01_TH.jpg]

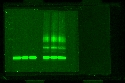

Supplement: Figure 6—source data 1. [file elife-91861-fig6-data1.zip › 0003578_01/0003578_01_TH.jpg]

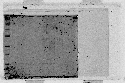

Supplement: Figure 6—source data 2. [file elife-91861-fig6-data2.zip › 0003631_01/0003631_01_TH.jpg]

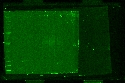

Supplement: Figure 6—source data 3. [file elife-91861-fig6-data3.zip › 0003633_01/0003633_01_TH.jpg]

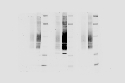

Supplement: Figure 7—source data 1. [file elife-91861-fig7-data1.zip › 0002306_02/0002306_02_TH.jpg]

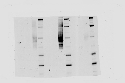

Supplement: Figure 7—source data 2. [file elife-91861-fig7-data2.zip › 0002307_02/0002307_02_TH.jpg]

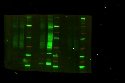

Supplement: Figure 7—source data 3. [file elife-91861-fig7-data3.zip › 0002305_02/0002305_02_TH.jpg]

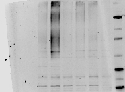

Supplement: Figure 7—source data 4. [file elife-91861-fig7-data4.zip › 0002883_07/0002883_07_TH.jpg]

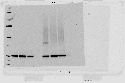

Supplement: Figure 8—source data 1. [file elife-91861-fig8-data1.zip › 0004178_02/0004178_02_TH.jpg]

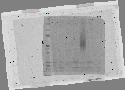

Supplement: Figure 8—source data 2. [file elife-91861-fig8-data2.zip › 0003092_03/0003092_03_TH.jpg]

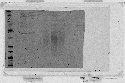

Supplement: Figure 8—source data 3. [file elife-91861-fig8-data3.zip › 0004210_01/0004210_01_TH.jpg]
